# Career choice in primary care: pre- and post-comparison of Honduran physicians completing social service

**DOI:** 10.26633/RPSP.2017.146

**Published:** 2017-12-05

**Authors:** E. Benjamín Puertas, Yoséf S. Rodríguez, E. Mariela Alvarado, Yolany Villanueva, Eyvilin Velasquez, Brian M. Erazo, Héctor Alfaro, Cheny Ortiz Dolmo

**Affiliations:** 1 Pan American Health Organization, Country Office Pan American Health Organization, Country Office Tegucigalpa Honduras Pan American Health Organization, Country Office, Tegucigalpa, Honduras.; 2 Secretary of Health of Honduras Secretary of Health of Honduras Tegucigalpa Honduras Secretary of Health of Honduras, Tegucigalpa, Honduras.; 3 National Autonomous University of Honduras National Autonomous University of Honduras Tegucigalpa Honduras National Autonomous University of Honduras, Tegucigalpa, Honduras.

**Keywords:** Primary health care, specialization, education, medical, graduate, Honduras, Atención primaria de salud, especialización, educación de posgrado en medicina, Honduras, Atenção primária à saúde, especialização, educação de pós-graduação em medicina, Honduras

## Abstract

**Objective.:**

*To describe and compare patterns of specialty choice among physicians in Honduras before and after completing mandatory social service; and to identify and compare salary perceptions and factors that may influence their career choice*.

**Methods.:**

*A quantitative methods approach was used on a cross-sectional questionnaire survey applied to 106 physicians completing social service in September 2015. Statistical analysis was performed using chi-square and factor analysis*.

**Results.:**

*Interest in family medicine was low and declined from 2.2% before social service to 0.9% after. Median annual expected income was 19.5% lower overall compared to the beginning of social service, and in particular, for primary care specialties (US$ 17 733), it was significantly lower than for other specialties (US$ 27 281). Participants reported that the most important factors influencing career choice were: income potential (23.3%), making a positive difference in people’s lives (19.4%), challenging work (10.7%), and perceived prestige (7.8%). Two factors were significantly associated with a preference for specialties other than primary care: the opportunity to teach (P= 0.008) and “makes positive difference in people’s lives“ (P = 0.005). When all categories were compared, “makes positive difference in people’s lives“ (P = 0.043), and opportunities to practice with independence (P = 0.036) were factors significantly associated to career decision*.

**Conclusion.:**

*Since interest in primary care among physicians decreased during social service and salary perception increased in favor of non-primary care careers, offering greater incentives for this specialty should be explored. Additional research to identify factors that might support the choice of a career in primary care are recommended*.

## RESUMEN

**Objetivo.:**

Describir y comparar los patrones de elección de especialidad de los médicos hondureños antes y después de completar el servicio social obligatorio. Establecer y comparar las percepciones sobre el salario y los factores que pueden influir en esa elección.

**Método.:**

Se aplicaron métodos cuantitativos a un cuestionario transversal administrado a 106 médicos que terminaron el servicio social en septiembre de 2015. El análisis estadístico se realizó mediante ji cuadrado y análisis factorial.

**Resultados.:**

El interés por la medicina familiar era escaso y descendió de 2,2% antes del servicio social a 0,9% después. La mediana de ingresos anuales previstos fue, en general, 19,5% inferior que al comienzo del servicio social y, en particular, fue significativamente inferior para las especialidades de atención primaria (US$ 17 733) que para otras especialidades (US$ 27 281). Los participantes informaron que los factores más importantes que influían en la elección de especialidad eran: posibles ingresos (23,3%), posibilidad de cambiar para mejor la vida de las personas (19,4%), trabajo estimulante (10,7%) y prestigio que pudiera otorgar (7,8%). Dos factores se asociaron significativamente con la preferencia por una especialidad fuera de la atención primaria: la oportunidad de enseñar (P= 0,008) y la posibilidad de “cambiar para mejor la vida de las personas“ (P = 0,005). Cuando se compararon todas las categorías, “cambiar para mejor la vida de las personas“ (P = 0,043) y la oportunidad de ejercer de forma independiente (P = 0,036) fueron factores que se asociaron significativamente con la decisión sobre la especialidad.

**Conclusión.:**

Como el interés por la atención primaria entre los médicos disminuyó durante el servicio social y la percepción sobre el salario inclinó la balanza en favor de especialidades distintas a la atención primaria, se debe explorar la posibilidad de ofrecer mayores incentivos para esta especialización. Se recomienda realizar nuevas investigaciones para determinar los factores que podrían apoyar la elección de la especialidad de atención primaria.

**Objetivo.:**

Descrever e comparar os padrões de escolha da área de especialidade entre médicos hondurenhos antes e depois da prestação do serviço social obrigatório e identificar e comparar a percepção salarial e de outros potenciais determinantes da escolha profissional.

**Métodos.:**

Estudo transversal com metodologia quantitativa conduzido com a aplicação de um questionário em uma amostra de 106 médicos que concluíram a prestação do serviço social obrigatório em setembro de 2015. A análise estatística foi realizada com o uso do teste de qui-quadrado e análise fatorial.

**Resultados.:**

Verificou-se baixo grau de interesse em medicina da família, com redução do interesse de 2,2% a 0,9% após o serviço social. Houve, em geral, uma redução de 19,5% na mediana anual prevista de remuneração comparada ao início do serviço social, sendo particularmente significativa nas especialidades de atenção primária (US$ 17.733) e relação às outras especialidades (US$ 27.281). Os participantes informaram os seguintes principais determinantes da escolha da carreira profissional: ter potencial de remuneração (23,3%), contribuir para melhorar a vida das pessoas (19,4%), ser um trabalho estimulante (10,7%) e prestígio percebido (7,8%). Observou-se uma associação significativa entre os seguintes fatores e a preferência por especialidades fora da atenção primária: oportunidade de ensinar (P = 0,008) e contribuir para melhorar a vida das pessoas (P = 0,005). Quando se compararam todas as categorias, contribuir para melhorar a vida das pessoas (P = 0,043) e oportunidade de exercer a medicina de forma autônoma (P = 0,036) foram os fatores significativamente associados à escolha da carreira.

**Conclusão.:**

Diante da perda de interesse por parte dos médicos pela área de atenção primária durante o serviço social e uma percepção salarial maior favorecendo carreiras fora da atenção primária, deve ser dado maior incentivo para que os profissionais optem por esta especialidade. Recomenda-se conduzir outros estudos para identificar os fatores que poderiam estimular a escolha de uma carreira profissional em atenção primária.

There is a shortage of primary care (PC) physicians in most countries in Latin America. For instance, the proportion of general practitioners (GPs) to specialists in Chile declined from 8 GPs of 10 specialists in 1996 to 6 of 10 in 2004 (1). PC physicians are directly related to the quality of PC services, and therefore, their numbers are a key factor in ensuring adequate coverage by high quality services. In 2011, Colombia had 1 family practitioner per 138 688 inhabitants (2), while Peru had 1 family practitioner per 267 324 inhabitants; however, the ideal ratio has been calculated to be 1 per 2 000 inhabitants (3).

Medical students still prefer hospital-related specialties. The proportion of Canadian medical school graduates who made family medicine their first option dropped from 40% in 1982 to only 28% in 2005 (4), while only one-third of medical students were interested in entering a family medicine program (5). In developing countries, the career goals of medical students are not aligned with the needs of the national health systems. For example, Peru opened 332 residency positions in family medicine in 2013 to address a shortage of 606 family practitioners, but had only 179 applicants (54%). There are several barriers that explain this phenomenon, including lower incomes for PC physicians versus specialists (6 – 9 – 6 – 9); less prestige given to PC careers (10, 11); and several aspects involving medical school training, such as PC being poorly reflected in the curricula, lack of exposure to family practitioners, negative perceptions of family doctors, and the prevailing negative culture towards PC (12). A systematic review in high-, middle-and low-income countries (13) found several factors that influence career choice in all countries: exposure to rural location, role models, and working conditions (facilitators); and low income, prestige, and medical school environment (barriers). Some factors were specific to middle-and low-income countries: understanding of rural needs and intellectual challenge. Other factors were specific to high-income countries: attitude towards social problems, voluntary work, influence of family, and length of residency.

Honduras is one of the five countries with the lowest density of human resources in health in the Region of the Americas (14). In 2015, there were 9 437 physicians (11 per 10 000 population), and only 12 family practitioners (0.127% of total medical workforce) (15). In 2015, the Secretary of Health reported a general practitioner to specialist ratio of 1 : 0.7. This gap could widen if the annual 4.5% growth rate of specialists continues, compared to just 2.9% for general practitioners (16). Honduras recently approved a National Health Plan for 2014 – 2018 that will require more PC physicians to lead 500 family health teams by 2017.

Little is known about factors that influence medical students in Honduras to choose primary care careers and the impact of social service in this decision. The specific research aims of this study were to (a) describe and compare patterns of specialty choice before and after completion of social service; and (b) identify and compare salary perception and factors that influence career choice in primary care before and after completion of social service.

## MATERIALS AND METHODS

### Study setting

Undergraduate medical education in Honduras consists of 8 years of study, the last of which is “Medical Social Service,“ a mandatory requirement for graduation. A lottery determines the location of each student’s 1 year of service, but in most cases it takes place within a primary care unit in a rural area or small town.

### Study design and sample

The quantitative method approach used in this study included a cross-sectional questionnaire applied to 106 Honduran medical students in their final year at either the *Universidad Nacional Autónoma de Honduras* (National Autonomous University of Honduras, Tegucigalpa, Honduras; UNAH) or the *Escuela Latinoamericana de Medicina* (Latin American School of Medicine, Havana, Cuba; ELAM). The study sample was composed of participants who completed an entry questionnaire at the start of their 1-year of social service in September of 2014 and an exit questionnaire at the end, in September 2015. Both questionnaires were the same. Subjects were identified during an orientation prior to graduation. Participants from the *Universidad Católica de Honduras* (Catholic University of Honduras, Tegucigalpa, Honduras) were not included because they were not present at the orientation.

### Survey instrument

A questionnaire was adapted from a survey (17) developed by the University of Alberta (Edmonton, Alberta, Canada) and translated into Spanish. It was used both before and after social service and addressed four main areas: demographic information, preferred medical specialties, salary perception, and factors influencing preferred specialty choice. Using a modified Likert scale ranging from 1 (very unimportant) to 5 (very important), participants rated the importance of each of 26 factors on their career choice. The list of medical career choices was adapted from lists used by the Canadian Resident Matching Service (Ottawa, Ontario, Canada); the developers of the FutureDocs Forecasting Tool (18); and the *Colegio Médico de Honduras* (Medical College of Honduras; Tegucigalpa, Honduras).

The questionnaires were administered at the UNAH School of Medicine at the beginning of the medical social service (September 2014), and following the participants’ completion of the program, during an orientation prior to graduation (September 2015). The investigators obtained oral consent from students who agreed to participate; all questionnaires were completed anonymously.

### Data analysis

Frequency distributions and percentages were calculated; the statistical analysis included chi-square test and Fisher’s exact test to assess relationships between two categorical variables belonging to a nominal or ordinal scale with *P* = 0.05. Statistical analysis was performed using IBM SPSS Statistics software, version 23 (SPSS Inc., an IBM company, Chicago, Illinois, United States).

## RESULTS

The number of completed surveys was 106, which represented 38.3% of the 277 physicians who completed their medical social service requirement during the study period; and 100% of those present at the time of the survey. Participants represented 45.3% of the original cohort that participated in the entry questionnaire. The majority (84%) of participants was more than 25 years of age, a significant increase over the prior year (59.4%; *P* = <0.001).

The number of female participants (*n* = 61; 58.7%) was greater than the number of male participants (*n* = 43; 41.3%), results that are consistent with the entry questionnaire. Most of the respondents considered themselves to be of mestizo origin (*n* = 93; 87.7%); racial background was similar for males and females.

Marital status of participants changed significantly by the end of the study period (*P* = 0.029), with more being married or living with a partner (27.4%) than at the onset (17.5%). The number of participants with no children decreased from 84% to 74%.

The majority of participants (88%) came from urban areas; rural origin increased from 8% to 11% among participants in the exit survey. The percentage of participants with a parent who is a medical doctor—in most cases, the father (77.8%)—decreased from 15% to 9.4%.

### Preferred medical specialties

The medical specialties most likely to be selected by participants were: gynecology/obstetrics (19.8%); surgery (10.4%); internal medicine, pediatrics, or dermatology (7.5%); traumatology and orthopedics (5.7%); and plastic surgery (4.7%). There was only one participant (0.9%) who selected family medicine, and another who selected general practice (0.9%). No one chose public health, nor tropical/infectious diseases. Only 20.5% students considered their response to be their final decision.

When compared to the results of the entry survey, interest in gynecology/obstetrics had almost doubled, from 9.8% at the onset of social service to 19.8% at the end. Interest in dermatology also increased, from 4.7% to 7.5%; while preference for plastic surgery almost doubled, from 2.6% to 4.7%. On the other hand, there was a general decline in the interest in primary care: family medicine declined from 2.2% to 0.9%; public health from 1.1% to 0%; and tropical and infectious diseases from 0.5% to 0%. Only general practice showed a slight increase in interest, from 0% to 0.9% (Table 1).

**FIGURE 1.**
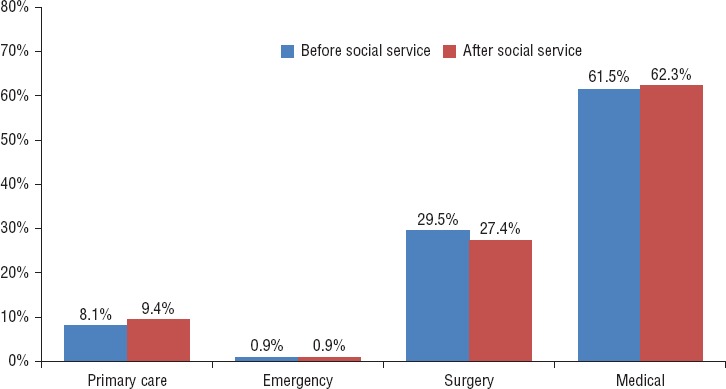
Preferred career choice of physician before and after completion of mandatory 1 year of medical social service, by specialty category (primary care, emergency, surgery, and medical), Honduras, September 2014–S2015

***Source:*** Prepared by the authors from study data

Specialties were recoded into four categories: 1) primary care, 2) emergency medicine, 3) surgery, and 4) medical. Primary care specialties included family medicine, general practice, pediatrics, and public health. Of the 106 respondents, 10 (9.4%) selected primary care specialties, compared to 8.1% on the entry survey (Figure 1). There was no significant difference between the entry and exit results with regard to emergency medicine, surgery, and medical specialties.

The researchers applied chisquare to test for homogeneity and independence of variables. As shown in Table 2, when related to specialty categories, the variables age, sex, and having a parent who was a medical doctor were statistically significant (*P* = 0.048, *P* = 0.001, and *P* = 0.016, respectively).

At the end of the year of social service, male participants showed a greater preference for surgery (46.5%), while females preferred medical specialties (78.7%). In the entry survey, males reported a preference for medical (49%) and surgical (42%) specialties, while women preferred medical (68%). Interest in PC slightly increased among females from 8% to 10% and remained the same in men (9%). When researchers compared the same specialty category between males and females, the women’s preference for primary care (60%) and medical specialties (73%) was higher than that of men (40% and 27%, respectively). More respondents from urban backgrounds preferred medical and surgical specialties (59.8% and 29.3%, respectively), while most participants from rural background preferred medical specialties (76.9%). There were more respondents from urban backgrounds who preferred primary care specialties at the exit study (9.8%) than upon entry (7.9%). Prior to the onset of social service, 42.1% of participants from rural backgrounds preferred surgical specialties, compared to 15.4% at the end. At the beginning of social service, only 1.3% of respondents who chose primary care specialties were completely sure of their choice, compared to 19.2% of participants who selected other specialties. At the end of the program, 2.8% of participants interested in primary care were sure of their choice, compared to 35.8% of those who selected other careers.

**TABLE 1.** Comparison of most preferred specialties among 234 (entry survey) and 106 (exist survey) medical students performing a year of social service in Honduras, September 2014 – September 2015

Specialties	1-year medical social service					
	Entry survey			Exit survey		
	n	%	Ranking	n	%	Ranking
Obstetrics/gynecology	23	9.8	1	21	19.8	1
Surgery	21	9.0	2	11	10.4	2
Psychiatry	20	8.5	3	8	7.5	_6_a
Internal medicine	19	8.1	4	8	7.5	4
Pediatrics	16	6.8	5	8	7.5	5
Cardiology	11	4.7	6	2	1.9	13^a^
Dermatology	11	4.7	7	8	7.5	_3_b
Urology	10	4.3	8	1	0.9	27^a^
Pediatric non-surgical specialties	9	3.8	9	4	3.8	18^a^
Traumatology and orthopedics	9	3.8	10	6	5.7	_7_b
Pediatric surgical specialties	7	3.0	15	4	3.8	_9_b
Plastic surgery	6	2.6	16	5	4.7	_8_b
Endocrinology	3	1.3	19	3	2.8	10^a^
Primary care						
Family medicine	2	0.9	25	1	0.9	22^a^
General practice	0	0.0	35	1	0.9	23^b^
Public health	1	0.4	32	0	0.0	35^a^

a Decreased ranking

b Increased ranking

***Source:*** Prepared by the authors from study data.

### Salary perception

The mean annual-expected income among survey participants at the end of social service was US$ 27 818 and the median was US$ 27 281, a decrease of 20.2% and 19.5%, respectively, compared to expectations at onset. The median annual-expected income of primary care specialties (US$ 17 733) was 35% lower than the median expected income of other specialties (US$ 27 281), and 37.2% lower compared to the beginning of social service. Median expected-income of surgical specialties (US$ 32 737 per year) was the highest compared to other specialties, and 45.8% higher than primary care (US$ 17 733). Female respondents estimated the salary of family physicians and general practitioners to be less than US$ 1 400 per month (21.2% and 33.7%, respectively), compared to 8.7% and 24% of male respondents. In the entry survey, 40% of female and 19.5% of male participants considered the salary of a general practitioner to be less than US$ 1 400. There was no significant difference in the case of family physicians. The salaries of other PC-related specialties were perceived to be US$ 1 400 – US$ 2 820 per month.

There was a significant relationship between monthly salary perception and specialty categories (*P* < 0.001). At onset of social service, the perceived monthly salary of participants who selected non-PC careers was significantly higher for non-PC physicians than for general practitioners, family practitioners, and pediatricians (*P* < 0.001). The perceived salary of participants was significantly lower for general practitioners (*P* = 0.013).

Participants’ salary perception of specialties other than PC increased significantly in the US$ 1 400 – 2 820 range, from 44.4% at the beginning of social service to 64.1% at the end; however, in the US$ 2 820 – 4 000 range, it decreased from 29.5% to 16%.

### Factors influencing preferred career

**FIGURE 2.**
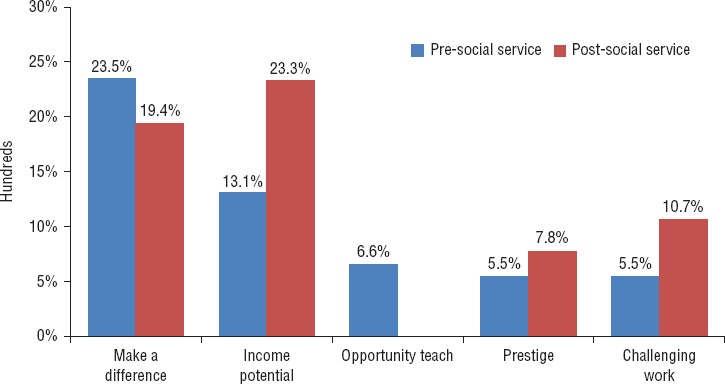
Most important factors in career choice among Honduran physicians: pre-and post-medical social service, 2014–2015

***Source:*** Prepared by the authors from study data.

As shown in Figure 2, participants considered the single most important factor influencing career choice to be: income potential (23.3%), making a positive difference in people’s lives (19.4%), challenging work (10.7%), and perceived prestige (7.8%). These were the same factors considered to be most important among participants beginning social service, with the exception of “opportunity to teach,“ which was selected more often at the end (6.6%). The exit survey also showed that income potential gained importance (23.3% vs. 13.1%). The opportunity to work with people with limited access to health care, to practice with professional independence, and to enjoy life outside of work were also important factors (4.9% each).

Two factors were significantly associated with a preference for specialties other than primary care: the opportunity to teach (84.4% vs. 50%; *P* = 0.008) and making positive difference in people’s lives (94.8% vs. 70%; *P* = 0.005), confirmed by the Fisher’s exact test (*P* = 0.020 and 0.027, respectively). When all categories were compared, making a difference in people’s lives“ (*P* = 0.043) and opportunities to practice with professional independence (*P* = 0.036) were factors significantly associated to career decisions among social service physicians (Table 3). Practice in ambulatory settings and preference for working in a rural community were not significant factors associated to PC career choice as they had been at onset. A factor that was not chosen in the entry survey proved to be influential among participants completing social service: opportunities to practice with professional independence.

When the category Emergency Medical Specialties was excluded (*n* = 1), opportunity to teach and making a difference in people’s lives continued to be factors significantly associated to non-PC career choice. When all categories were compared, “variety of medical problems“ (*P* = 0.032) and “predictable hours“ (*P* = 0.047) were statistically significant to career choice. These factors were different from those that influenced career selection at the beginning of social service.

## DISCUSSION

Primary care careers are not the first choice of specialty, neither before nor after the 1 year of medical social service required of Honduran students. Demographic characteristic did not change significantly, except marital status and having children. With few exceptions, the same pattern of specialty choice was maintained from entry to exit surveys. Primary care careers continued to be at the bottom of desirable options for the study sample. Moreover, social service did not motivate participants to pursue primary care careers. Participants continued to favor gynecology/obstetrics, surgery, and internal medicine, while specialties such as dermatology and plastic surgery were added to the top 10 choices. This could be attributed to the perception that medical and surgery specialists with hospital experience have a higher status than do primary care physicians; therefore, medical students sought a similar choice (19). The greater interest in obstetrics and gynecology could be due to the fact that social service allowed participants to apply related procedures and raised awareness of the higher demand for women-related health care services. Moreover, there is a larger offering of obstetric and gynecological residency positions in the country compared to other specialties. There is also the possibility that UNAH medical students had a higher interest in obstetrics and gynecology than did students at the Catholic University, which did not participate in the exit study.

**TABLE 2.** Characteristics of respondents in a study of career choice in primary care after social service, by preferred specialty category, Honduras, September 2015

Characteristics	Specialty									
	Primary care		Emergency medicine		Surgery		Medical		Total	
	n	%	n	%	n	%	n	%	n	%
University that conferred the medical degree										
UNAH^a^	9	10.1	1	1.1	25	28.1	54	60.7	89	100
ELAM^b^	1	6.7	0	0.0	3	20.0	11	73.3	15	100
No response	0	0.0	0	0.0	1	50.0	1	50.0	2	100
Total	10	9.4	0	0.9	29	27.4	66	62.3	106	100
Age^c^										
≤ 25 years	5	33.3	0	0.0	4	26.7	6	40.0	15	100
> 25 years	5	5.6	1	1.0	24	27.0	59	66.3	89	100
No response	0	0.0	0	0.0	1	50.0	1	50.0	2	100
Total	10	9.6	1	0.9	28	26.9	66	62.3	106	100
Sex^c^										
Male	4	9.3	1	2.3	20	46.5	18	41.9	43	100
Female	6	9.8	0	0.0	7	11.5	48	78.7	61	100
No response	0	0.0	0	0.0	2	100.0	0	0.0	2	100
Total	10	9.4	1	0.9	29	27.4	66	62.3	106	100
Race										
White	0	0.0	0	0.0	6	66.7	3	33.3	9	100
Mestizo	10	7.8	1	1.1	22	23.9	59	64.1	92	100
Afro-descendant	0	0.0	0	0.0	0	0.0	4	100.0	4	100
Indigenous group	0	0.0	0	0.0	1	100.0	0	0.0	1	100
Other	0	0.0	0	0.0	0	0.0	0	0.0	0	100
Total	10	9.4	1	0.9	29	27.4	66	62.3	106	100
Marital status										
Single	7	9.6	1	1.4	21	28.8	44	60.3	73	100
Married/common law	2	6.9	0	0.0	6	20.7	21	72.4	29	100
Separated/divorced/widowed	1	25.0	0	0.0	2	50.0	1	25.0	4	100
No answer	0	0.0	0	0.0	0	0.0	0	0.0	0	100
Total	10	9.4	1	0.9	29	27.4	66	62.3	106	100
Location/area where student lived prior to university										
Rural	1	7.7	1	1.1	2	15.4	10	76.9	13	100
Urban	9	9.8	0	0.0	27	29.3	55	59.8	92	100
No response	0	0.0	0	0.0	0	0.0	1	100.0	1	100
Total	10	9.5	1	1.0	29	27.6	66	62.3	106	100
Parent is a physician^c^										
Yes	0	0.0	0	0.0	7	77.0	3	30.0	10	100
No	10	10.4	1	1.0	22	22.9	63	65.6	96	100
Total	10	9.4	1	0.9	29	29.5	66	62.3	106	100

a *Universidad Nacional Autónoma de Honduras* (National Autonomous University of Honduras), Tegucigalpa, Honduras.

b *Escuela Latinoamericana de Medicina* (Latin American School of Medicine), Havana, Cuba.

c Statistically significant (*P* < 0.05).

***Source:*** Prepared by the authors from study data.

When re-coded into four specialty categories, interest in primary care careers increased slightly after social service (9.4%), although it was lower than figures reported by studies elsewhere (1, 20 – 24 – 20 – 24). Most of the interest in PC was attributable to pediatrics, a specialty classified as a primary care career for the purpose of this study, though pediatricians and physicians in Honduras do not consider it as such. Final choice of a primary care specialty might be even lower, since only 2.8% of these participants were completely certain; still, the exit survey showed an increase over the 1.3% of the entry survey. On the other hand, 35.8% were certain of a non-PC specialty upon exit.

The uncertainty regarding PC careers is an opportunity for the Secretary of Health and the UNAH to motivate students to pursue the PC specialties. This study reaffirms known trends governing student career choice in Honduras, i.e., making a positive difference in people’s lives, and it adds other influential factors, such as the opportunity to teach and to practice with professional independence. The study also found no significant difference between men and women who preferred primary care. Most reviewed evidence (5, 10, 21, 25 – 27) found a female predominance in preference for primary care careers.

Choice of medical specialty continued to be based on a combination of ambition and prestige on one hand, and on personal and altruistic considerations on the other. Padilla (28) pointed out that motivation to specialize in Costa Rica had an altruistic component, mostly related to self-determination rather than economic interests, results that counter those found in Honduras where income potential was the main factor. At completion of social service, participants considered “income potential“ as the most important factor in career choice, ranking higher than at the year’s onset. However, two intrinsic factors absent from the top-5 list at the entry point became influential among participants as reflected by the exit survey: “working with vulnerable population“ and “opportunity to enjoy life outside work.“ This could mean that proximity to communities and people with inadequate access to health care influenced some participants.

There were no factors significantly associated to PC career choice at the end of social service. Those factors that were facilitators for selecting PC careers prior to social service (“practice in ambulatory settings“ and “preference for working in a rural community“) were not influential by the end. It could mean that the experience of social service did not motivate participants to select primary care as a career, or that it even undermined any desire to pursue PC. However, there were other variables that could have had a negative influence on PC careers, e.g., delays in due payments, lack of medical supplies, lack of supervision, among others. This would be consistent with findings in Peru, where deficiencies and limitations in rural health care reduced physicians’ intentions to work in those areas (29).

Two factors were significantly associated to non-PC career choice on the exit survey: opportunity to teach and making a positive difference in people’s lives. This is consistent with findings among medical students from Peru, whose vocation relates to helping people and having a will to serve them, although those intentions could change at the end of their career when they are confronted with the labor market (30). Most medical training during the final years takes place in a hospital, thus focusing on specialties; almost no primary care training is performed at the hospital level.

Annual salary perception decreased during the 1-year social service period. This reduction could be related to the real-life experience of working in a community setting, a context quite different from the academic environment. Additionally, interaction with other colleagues could have influenced this perception. Even so, annual salary perception was still higher than physicians’ salaries in Honduras at the time of the study. Expected income of PC specialties compared to other specialties was even lower than at the onset, although in reality, it differs by only US$ 248 per month in Honduras. This difference is almost one-half the difference perceived by respondents in the entry survey (US$ 471) and one-third of the difference perceived at the exit (US$ 795). This is unique in Latin America where salaries for specialists are usually much higher. Higher salary perceptions for medical and surgical specialties will continue favoring those specialties.

Female participants perceived PC salaries to be lower than did their male counterparts, and was even lower by the exit survey. Since evidence suggests that women are more inclined to choose PC careers, this could be an additional barrier in Honduras where potential income was shown to be the most influential factor in career selection.

### Limitations

The cross-sectional nature of this study makes it difficult to explain career interest in PC at different stages of medical training; therefore, the findings might not be generalizable to all undergraduate students. The exit study did not reach students at the Catholic University, thus the results could have a bias towards the preferences of UNAH participants. The questionnaire measured the “preferred“ choice of medical career, and not the “actual“ decision. The small number of respondents preferring PC and emergency medicine specialties limited the possibility of further analysis.

## Conclusions

Interest in primary care careers did not increase by the end of the 1-year medical social service required of physicians in Honduras. In fact, there were several indications that it decreased. Although the social service program provided an opportunity for exposure to ambulatory settings and work in rural areas, it could be failing to motivate graduates toward PC careers; its approach to primary care needs to be reinforced. Salary perception became more realistic toward the end of social service, but the change favored non-primary care careers. The study identified factors that facilitate career choice in primary care, surgery, and medical specialties. However, at the end of social service it did not identify factors that could influence career choice toward primary care.

**TABLE 3.** Factors influencing physicians’ career choices at the end of social service, by specialty category, Honduras, September 2015

Factor	Specialty category											
	Primary care (n=10)		Emergency Medicine (n=1)		Surgery (n=29)		Medical (n=66)		All specialties combined (n=96)		All specialties compared	Primary care specialties compared to all other specialties combined
	n	%	n	%	n	%	n	%	n	%	P	P
Income potential	6	60	1	100	24	82.3	50	75.8	75	78.1	0.481	0.199
Perceived prestige	6	60	1	100	25	86.2	51	77.3	77	80.2	0.34	0.14
Opportunity to teach	5	50	1	100	24	82.8	56	84.8	81	84.4	0.066	0.008^a^
Preference for working in a rural community	4	40	0	0	7	24.1	32	48.5	39	40.6	0.13	0.969
Preference for working in an urban center	6	60	0	0	17	58.6	47	71.2	64	66.7	0.307	0.672
Preference/influence of family, friends or community	3	30	0	0	13	44.8	31	47	44	45.8	0.702	0.471
Makes a positive difference in people’s lives	7	70	1	100	27	93.1	63	95.5	91	94.8	**0.043**^**a**^	**0.005**^**a**^
Perceived intellectual content of discipline	8	80	1	100	26	89.7	60	90.9	87	90.6	0.746	0.294
Opportunity for research	7	70	1	100	23	79.3	62	93.9	77	80.2	0.843	0.449
Opportunity to work on highly challenging cases	9	90	1	100	28	96.6	62	93.9	91	94.8	0.874	0.533
Opportunity to work on acute medical problems	10	100	1	100	26	89.7	61	92.4	89	92.7	0.873	0.758
Emphasis on continuity of care of patients	10	100	1	100	25	86.2	62	93.9	88	94.6	0.383	0.452
Opportunity to deal with a variety of medical problems	9	90	1	100	22	75.9	61	92.4	84	87.5	0.07	0.235
Early exposure to the discipline	9	90	1	100	23	79.3	58	87.9	82	85.4	0.671	0.692
Opportunity to work with people with limited access to health care	9	90	1	100	23	79.3	59	89.4	83	86.5	0.563	0.753
Length of residency	6	60	1	100	14	48.3	44	67.7	59	62.1	0.279	0.896
Ability to use a wide range of skills & knowledge in patient care	9	90	1	100	29	100	61	93.8	91	95.8	0.497	0.414
Ability to master a small set of skills and be the “expert“	8	80	0	0	20	69	45	68.2	65	67.7	0.423	0.424
A positive interaction with a clinician/teacher of this specialty	9	90	1	100	24	82.8	56	84.8	81	84.4	0.922	0.636
Current debt load to study medicine	2	20	0	0	7	24.1	26	40	33	34.7	0.336	0.448
More leisure time	0	0	0	0	2	7.1	8	12.1	10	10.5	0.602	0.281
Opportunities to practice with professional independence	8	80	0	0	22	75.9	59	89.4	81	84.4	**0.036**^**a**^	0.719
Emphasizes practice in ambulatory settings	4	40	0	0	11	39.3	42	63.6	53	55.8	0.076	0.34
Predictable work hours	4	40	0	0	13	46.4	45	69.2	58	61.7	0.055	0.184
Provides an opportunity to enjoy life outside of work	6	60	1	100	17	60.7	48	73.8	66	70.2	0.485	0.506
Development of long-term relationships with patients	6	60	1	100	18	62.1	51	78.5	70	73.7	0.273	0.357

a *P* < 0.05

***Source:*** Prepared by the authors from study data.

**FIGURE 3:**
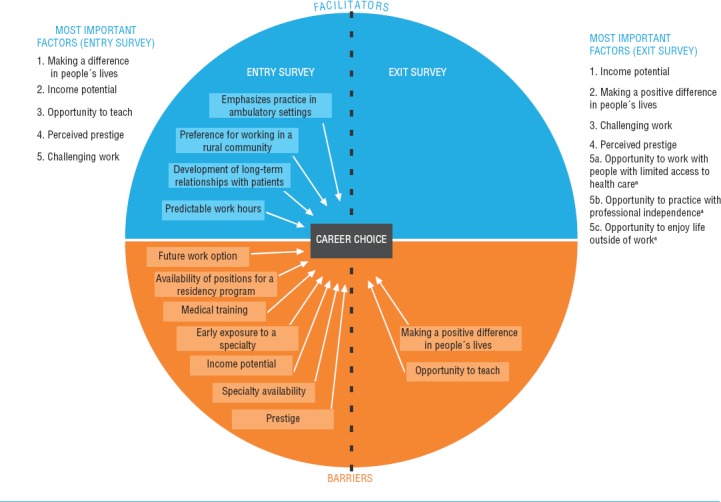
Factors influencing medical students’ choice of primary care as a career before and after completion of social service, Honduras, September 2014–2015.

^*^ These three factors shared 5th place.

Since several countries in the Region have a social service requirement to practice medicine and a shortage of primary care physicians, ministries of health and schools of medicine should improve the experience, perhaps by offering monetary compensation, follow-up, professional development, job supplies, mentoring and tutoring, among others. More research is needed to understand what incentives could influence more physicians to choose primary care.

## Acknowledgements

The authors appreciate the support of the *Universidad Nacional Autónoma de Honduras* and its authorities. They also wish to express their gratitude to Katherine Senter for her editing, to Ana Treasure for advocating with national authorities to present these data, and to all study participants.

## **Disclaimer**.

Authors hold sole responsibility for the views expressed in the manuscript, which may not necessarily reflect the opinion or policy of the *RPSP/PAJPH* and/or PAHO.
